# *N*,*N*-Dimethyldithiocarbamate Elicits Pneumococcal Hypersensitivity to Copper and Macrophage-Mediated Clearance

**DOI:** 10.1128/iai.00597-21

**Published:** 2022-03-21

**Authors:** Sanjay V. Menghani, Madeline P. Cutcliffe, Yamil Sanchez-Rosario, Chansorena Pok, Alison Watson, Miranda J. Neubert, Klariza Ochoa, Hsin-Jung Joyce Wu, Michael D. L. Johnson

**Affiliations:** a Department of Immunobiology, University of Arizona College of Medicine—Tucson, Tucson, Arizona, USA; b Medical Scientist Training M.D.-Ph.D. Program (MSTP), University of Arizona College of Medicine—Tucson, Tucson, Arizona, USA; c Arizona Arthritis Center, University of Arizona College of Medicine—Tucson, Tucson, Arizona, USA; d Valley Fever Center for Excellence, University of Arizona College of Medicine—Tucson, Tucson, Arizona, USA; e BIO5 Institute, University of Arizona College of Medicine—Tucson, Tucson, Arizona, USA; f Asthma and Airway Disease Research Center, University of Arizona College of Medicine—Tucson, Tucson, Arizona, USA; New York University School of Medicine

**Keywords:** *Streptococcus pneumoniae*, dimethyldithiocarbamate, DMDC, macrophages, antibiotic, copper, copper-dependent toxicity, antimicrobial activity, antimicrobial combinations, flow cytometry, metal

## Abstract

Streptococcus pneumoniae is a Gram-positive, encapsulated bacterium that is a significant cause of disease burden in pediatric and elderly populations. The rise in unencapsulated disease-causing strains and antimicrobial resistance in S. pneumoniae has increased the need for developing new antimicrobial strategies. Recent work by our laboratory has identified *N*,*N*-dimethyldithiocarbamate (DMDC) as a copper-dependent antimicrobial against bacterial, fungal, and parasitic pathogens. As a bactericidal antibiotic against S. pneumoniae, DMDC’s ability to work as a copper-dependent antibiotic and its ability to work *in vivo* warranted further investigation. Here, our group studied the mechanisms of action of DMDC under various medium and excess-metal conditions and investigated DMDC’s interactions with the innate immune system *in vitro* and *in vivo*. Of note, we found that DMDC plus copper significantly increased the internal copper concentration, hydrogen peroxide stress, nitric oxide stress, and the *in vitro* macrophage killing efficiency and decreased capsule. Furthermore, we found that *in vivo* DMDC treatment increased the quantity of innate immune cells in the lung during infection. Taken together, this study provides mechanistic insights regarding DMDC’s activity as an antibiotic at the host-pathogen interface.

## INTRODUCTION

Streptococcus pneumoniae (the pneumococcus) is a Gram-positive bacterium that is typically carried asymptomatically in the nasopharynx in the pediatric population (1). S. pneumoniae is a significant cause of acute otitis media, community-acquired pneumonia, bacterial sepsis, and meningitis (2). Despite the increased utilization of the pneumococcal conjugate vaccine (PCV), there is still a significant disease burden globally, contributing to 3.7 million episodes of severe pneumococcal disease and up to 515,000 pediatric deaths due to pneumococcal disease in 2015 (3). In a recent study of children in northern Ghana, postvaccination carriage of S. pneumoniae was dominated by serotypes not covered by the vaccine (4). This disease burden is not limited to the pediatric population, as the pneumococcus was responsible for over 494,000 deaths in the elderly (those over 70 years old) in 2016 (5). Further studies have shown increases in strains not covered by the conjugate vaccine of S. pneumoniae causing invasive disease (6). In addition, the threat of antibiotic resistance in S. pneumoniae creates a further impetus to develop novel antimicrobial strategies (7). The U.S. Centers for Disease Control and Prevention identified drug-resistant S. pneumoniae as a serious threat in their 2019 report *Antibiotic Resistance Threats in the United States* (8).

There is strong evidence for the interplay between the pneumococcus and the innate immune system. The pneumococcus is engulfed and destroyed by phagocytes, including macrophages and neutrophils (9). There is evidence for the cooperative bactericidal activity of these two innate immune cell populations (10). Neutrophils degrade phagocytosed S. pneumoniae via serine proteases like neutrophil elastase and cathepsin G (CG) (11). Neutrophil depletion has been shown to ablate pneumococcal clearance rates in a murine pneumonia model (12). Macrophage LC3-associated phagocytosis (LAP) is an established immune defense that contributes to S. pneumoniae clearance (13). LAP-mediated bacterial killing is diminished in aged mice compared to young mice (13). Alveolar macrophages also utilize apoptosis-associated bacterial killing to prevent murine pneumonia (14). The macrophage phagolysosome uses several mechanisms to clear engulfed pathogens, including hydrogen peroxide stress, nitric oxide stress, low pH, activated proteases like lysozyme, and copper intoxication (15). Accordingly, the pneumococcus has evolved to utilize a copper efflux ATPase, CopA, to efflux increased intrabacterial copper to antagonize macrophage-mediated copper intoxication (16). These innate immune cell types are crucial for controlling S. pneumoniae infection in the human host (17).

The pneumococcus produces several additional factors that contribute to innate immune cell evasion. The pneumococcus produces neutrophil elastase inhibitors that reduce damage to the lung caused by neutrophils during pneumococcal pneumonia, thus decreasing inflammation and increasing bacterial survival (18). S. pneumoniae also produces the autolysin LytA, which contributes to capsule shedding and the release of the intracellular pneumolysin (Ply) (19). As a result of autolyzed bacterial contents being in the blood, phagocytosis of intact bacteria by peripheral blood mononuclear cells is impaired (20–22). Accordingly, there is also a substantial body of evidence to suggest that the pneumococcus may be more prone to infecting the elderly due to a decline in innate immune system activity with age (13, 23, 24).

At the host-pathogen interface, there is a battle for nutrients. As the host tries to restrict the pathogen’s access to available vitamins and nutrients, the pathogen is upregulating mechanisms to steal these necessary factors for growth. This battle, known as “nutritional immunity,” is a crucial component of the innate immune system for pathogenic clearance (25, 26). While the transition metals Ca, Mn, Zn, and Fe are necessary for pneumococcal growth, Cu and Zn are toxic in excess (27, 28). The human host innate immune system has developed strategies to sequester essential metal cofactors like Ca, Fe, Mn, and Zn, while pathogens have evolved mechanisms to counteract these sequestration strategies (29–32).

While toxic to some bacteria in large amounts, copper is well utilized and well regulated in humans. Copper dysregulation in humans is observed in the rare genetic conditions Wilson’s disease (hepatolenticular degeneration) and Menke’s disease (33, 34). *In vivo* mutation of the copper transporter ATP7A, the same transporter mutated in human Menke’s disease, has been shown to decrease the macrophage response to dermal wounds (35). Thus, among the many mechanisms that the phagolysosomes utilize, copper intoxication of engulfed pathogens for bactericidal activity is a vital yet understudied strategy. Copper generally increases in concentration in areas of infection (36, 37). For specific cells such as the macrophage, the ATP7A copper-transporting ATPase is a mediator for delivering copper into the phagolysosome, without which bacterial clearance is significantly compromised (38). Macrophage-activating cytokines like interferon gamma (IFN-γ) increase the phagolysosome’s copper levels, priming for bactericidal activity (38). Peritoneal macrophages of copper-deficient rats display impaired respiratory burst and fungicidal activity (39). There is a growing body of evidence to use increased Cu and Zn to directly intoxicate intracellular pathogens within phagocytes as an antibiotic mechanism (40). In 2014, Festa et al. developed a copper-dependent antimicrobial against the fungus Cryptococcus neoformans that increases the efficiency of macrophage fungicidal activity (41). The role of antibiotics interacting with macrophages is complex and antibiotic dependent (42). It is unclear whether most antibiotics in clinical use are taken up by macrophages and improve a macrophage’s inherent killing rate or aid in the clearance rate *post hoc* of antibiotic-poisoned pathogens (42).

Our laboratory recently identified *N*,*N*-dimethyldithiocarbamate (DMDC) as a compound that can serve as a copper-dependent antibiotic against the pneumococcus from a small-molecule screen of copper ionophore compounds (43). While additional copper ionophore compounds have been identified, the mechanisms of action are not very well understood (44–46). Here, we investigated the mechanism of DMDC activity as a copper-dependent antimicrobial at the host-pathogen interface. We demonstrated that DMDC is effective as an antibiotic in a nutrient-rich medium (M17 medium) and a medium traditionally used for culturing leukocytes to more closely mimic the host niche (Roswell Park Memorial Institute [RPMI] 1640) (47). We demonstrated that DMDC’s copper-dependent toxicity requires constant exposure and that cold-temperature treatment ablated DMDC’s killing effect. We also demonstrated that exposure to DMDC causes an increase in copper within the bacterial cell, further providing evidence for exacerbating mismetallation as a killing mechanism due to manganese’s ability to rescue DMDC’s copper-dependent toxicity. Additionally, we explored DMDC’s potential to aid in macrophage-mediated killing and subsequent clearance mechanisms, finding that increased zinc levels, hydrogen peroxide exposure, and nitric oxide exposure are potential mechanisms by which DMDC aids in macrophage-mediated killing. Furthermore, we demonstrated that combination treatment with DMDC and copper decreases S. pneumoniae capsule, a mechanism utilized by the bacterium to prevent opsonization and phagocytosis by macrophages. Finally, we demonstrated that DMDC increased macrophage, dendritic cell (DC), and neutrophil recruitment to the lung during infection. Taken together, our results provided insights into DMDC as a copper-dependent antibiotic that can work to aid in the innate immune clearance of S. pneumoniae.

## RESULTS

### DMDC is a copper-dependent antibiotic in nutrient-rich (M17) and host-niche-mimicking (RPMI 1640) media.

Our laboratory conducted a targeted small-molecule screen for compounds with ionophoric properties that can serve as a copper-dependent antibiotic against the pneumococcus (43). Additionally, our laboratory reviewed the compositions of growth media for S. pneumoniae, exposing heterogeny in the characteristics of bacteria grown in nutrient-rich media (like Todd-Hewitt broth [ThyB] and M17 media) in comparison to a more “minimal” host-niche-mimicking medium (like RPMI 1640) (48). A major distinguishing factor between the two nutrient-rich media (ThyB and M17) is that M17 is prepared and sterilized without a carbon source; the manufacturer suggests that a 10% lactose solution or an alternative carbon source can be added after sterilization to provide greater control over the medium composition for an investigator (48). We performed growth curves and killing curves to further examine this dichotomy by utilizing nutrient-rich M17 medium and host-niche-mimicking RPMI 1640, as RPMI 1640 is traditionally used for cell culture of lung epithelial cells and leukocytes (47, 49). *In vitro* growth curve assessment of TIGR4 S. pneumoniae in M17 medium supplemented with copper with or without DMDC demonstrated a significant growth defect observed with the combination of 500 μM Cu^2+^ plus 32 μM DMDC (see Fig. S1A in the supplemental material), which is equivalent to the combination required for toxicity seen previously in ThyB (43). Incubation under the combination condition obtained a significant decrease in CFU per milliliter to a level below our level of detection at the time point (*t*) of 120 min, indicating bactericidal activity for the 500 μM Cu^2+^ plus 32 μM DMDC combination (Fig. S1B). Our previous work identifying DMDC as a copper-dependent antibiotic showed *in vivo* efficacy in a murine pneumonia model (43). Since the compound has efficacy *in vivo*, we wanted to explore further how this compound works in a more nutrient-restricted and host-niche-mimicking medium, such as RPMI 1640. An *in vitro* growth curve assessment of TIGR4 S. pneumoniae in RPMI 1640 medium supplemented with copper with or without DMDC demonstrated a significant growth defect observed with the combination of 50 μM Cu^2+^ plus 16 μM DMDC (Fig. S1C). TIGR4 S. pneumoniae in RPMI 1640 medium supplemented with copper with or without DMDC in a killing curve demonstrated a bactericidal combination of 250 μM Cu^2+^ plus 16 μM DMDC at the 180-min and 240-min time points (Fig. S1D). For combinations of copper plus DMDC utilizing less than 250 μM Cu^2+^, no killing effect, bactericidal or bacteriostatic, was observed (data not shown). It is interesting to note that the time required for killing is longer in RPMI 1640 (3 to 4 h) than in M17 (1 to 2 h) (Fig. S1B and D). These data demonstrate that DMDC is a copper-dependent bactericidal antimicrobial in various growth media ranging from host-niche-mimicking to nutrient-rich media.

### DMDC’s bactericidal activity requires constant exposure and is temperature dependent.

**FIG 1:**
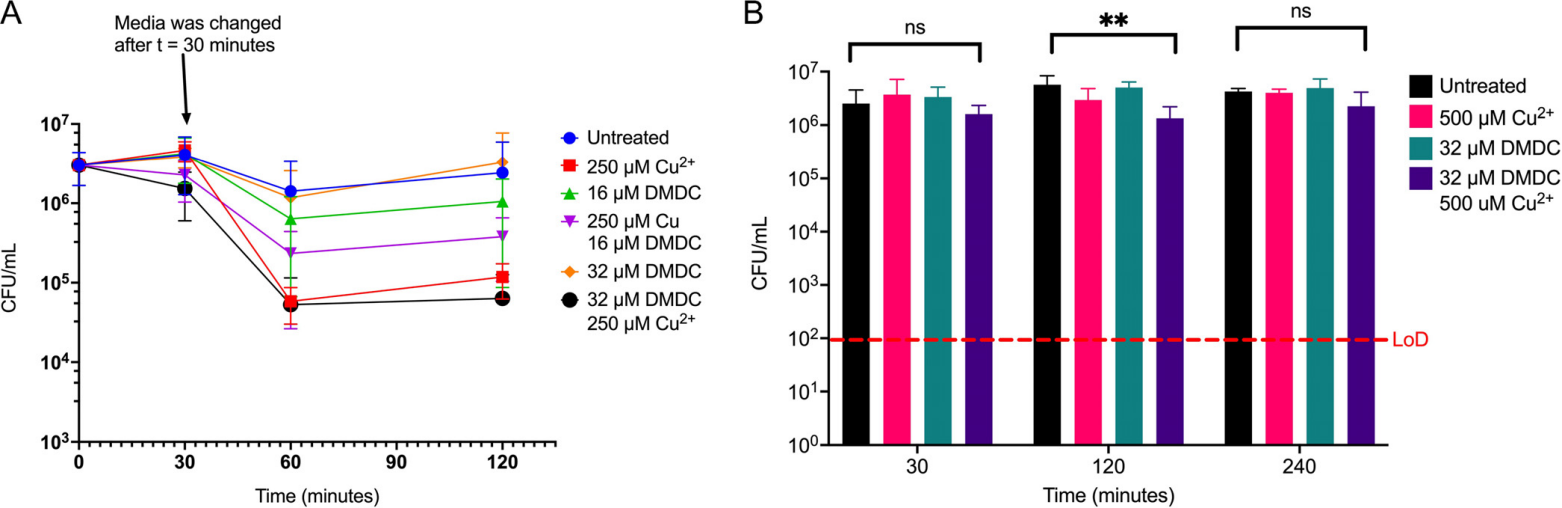
DMDC’s bactericidal activity requires constant exposure and is temperature dependent. (A) Killing curve of WT TIGR4 S. pneumoniae starting with an inoculum of 3.0 × 10^6^ CFU/mL in M17 medium supplemented with the indicated concentrations of copper and/or DMDC for 30 min before bacteria were pelleted and resuspended in fresh M17 medium without supplementation. The killing effect of 500 μM Cu^2+^ plus 32 μM DMDC is sustained, as the bacteria show evidence of static CFU counts over the next two time points, while the other conditions show recovery of growth between the 60-min and 120-min time points. (B) Killing curve of WT TIGR4 S. pneumoniae in M17 medium performed at 4°C. Starting with an inoculum of 4.4 × 10^6^ CFU/mL in M17 medium supplemented with the indicated concentrations of copper and/or DMDC, the killing effect of 500 μM Cu^2+^ plus 32 μM DMDC is ablated as there is no statistically significant difference of CFU counts between the untreated and combined conditions at the 240-min time point. All bars for killing curves represent means ± standard deviations (SD) (*n* = 9 across 3 independent replicates). Statistical differences were measured by Student’s *t* test (ns, not significant; *, *P* < 0.05; **, *P* < 0.01; ***, *P* < 0.001; ****, *P* < 0.0001).

We sought to further understand how DMDC is interacting with the bacterium. We hypothesized that the copper-dependent toxicity of DMDC is an irreversible process leading to bacterial death. To test this effect, TIGR4 S. pneumoniae bacteria were incubated in supplemented M17 medium for 30 min before pelleting and resuspending the bacteria in fresh medium lacking supplementation. While the pneumococcus continues to show decreased CFU per milliliter at the 60-min time point under the combinatory DMDC plus copper conditions, the bacterial counts remained static (Fig. 1A), as opposed to seeing continued killing at the 120-min time point in Fig. S1B.

As temperature is a factor contributing to active transport, with lower temperatures inhibiting active transport (50, 51), we extended this principle to perform a killing curve in an ice bath (4°C) to prevent DMDC from entering the cell. Without active transport, we hypothesized that the combination of copper plus DMDC would not be able to work as efficiently as a bactericidal antibiotic. At 4°C, the combination of 500 μM Cu^2+^ plus 32 μM DMDC no longer killed the pneumococcus as it did at 37°C (Fig. 1B and Fig. S1B). There is a statistically significant difference in bacterial CFU counts between untreated and combination conditions at the 120-min time point, but this significance is not present at the 240-min time point. Overall, these data show that DMDC’s copper-dependent toxicity requires constant exposure to a bacterium and that this toxicity is temperature dependent, indicating that this compound is likely actively transported into the bacterium.

### Exposure to copper plus DMDC causes an increase in intracellular copper.

**FIG 2:**
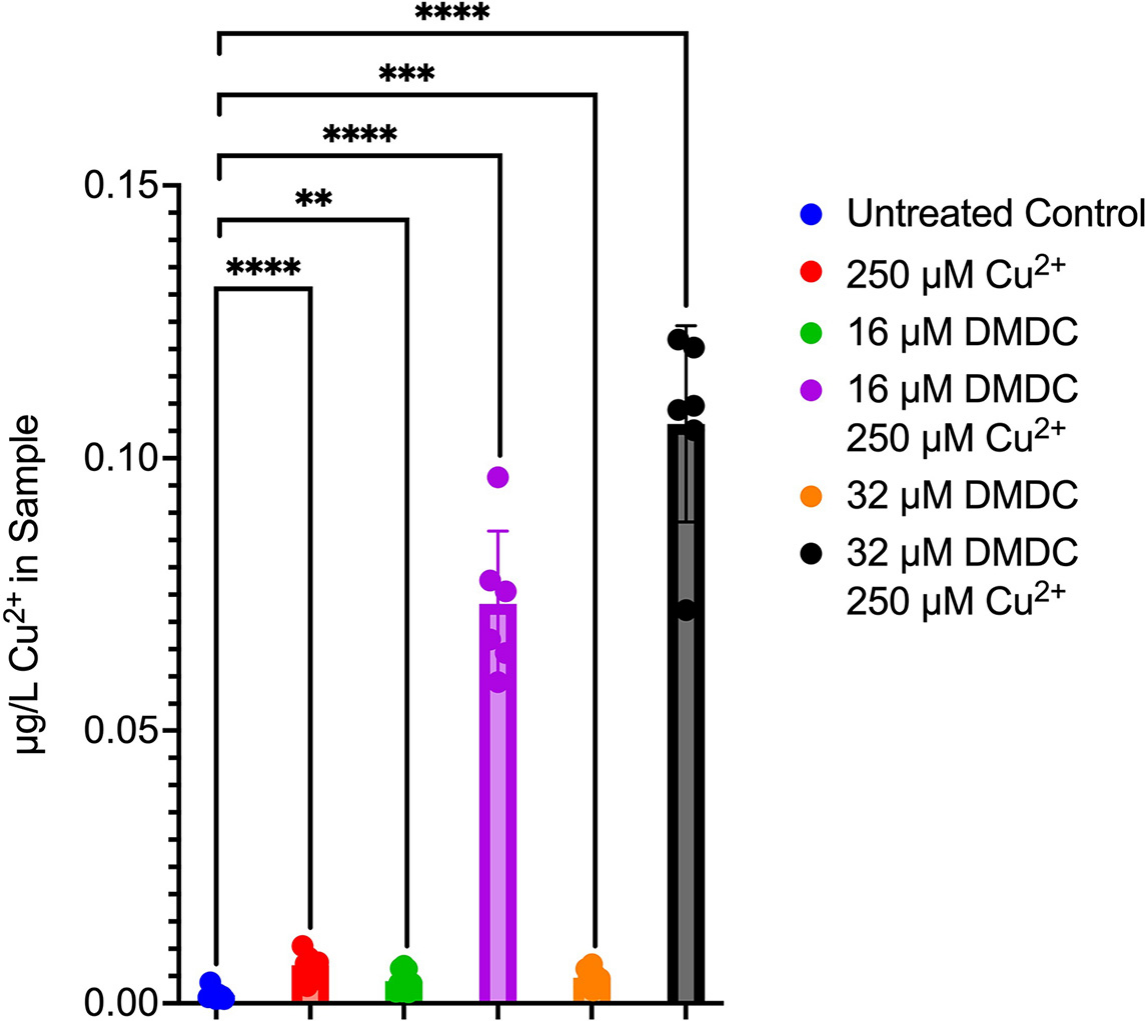
DMDC and copper treatment leads to a significant increase in intracellular copper. GFAAS analysis of bacterial pellets showed a marked statistically significant increase in the copper content within the bacteria treated with 250 μM Cu^2+^ plus 16 μM DMDC and within the bacteria treated with 250 μM Cu^2+^ plus 32 μM DMDC in comparison to the untreated control. Experiments were performed in triplicate, with statistical significances of differences determined by an unequal-variance *t* test (ns, not significant; *, *P* < 0.05; **, *P* < 0.01; ***, *P* < 0.001; ****, *P* < 0.0001).

To determine DMDC’s effect on copper intoxication of the pneumococcus, we quantified the concentration of copper within the bacterium after treatment with 250 μM Cu^2+^ with or without 16 or 32 μM DMDC. Compared to the untreated control, we show that there is a statistically significant increase in the copper content within the bacteria treated with 250 μM Cu^2+^ plus 16 μM DMDC and within the bacteria treated with 250 μM Cu^2+^ plus 32 μM DMDC (Fig. 2). Compared to the copper-treated samples, the 250 μM Cu^2+^- and 16 μM DMDC-treated bacteria and the bacteria treated with 250 μM Cu^2+^ plus 32 μM DMDC have 7- and 10-fold increases in intracellular copper, respectively.

To further investigate the concentrations of intrabacterial nutrient metals, we utilized inductively coupled plasma-optical emission spectroscopy (ICP-OES) to quantify the concentrations of zinc, manganese, copper, and calcium. Compared to the untreated control, we show that there is a statistically significant increase in the copper content within the bacteria treated with 250 μM Cu^2+^ plus 16 μM DMDC and within the bacteria treated with 250 μM Cu^2+^ plus 32 μM DMDC (Fig. S2A). Interestingly, the ICP-OES method determined that 250 μM Cu^2+^- and 16 μM DMDC-treated bacteria and the bacteria treated with 250 μM Cu^2+^ plus 32 μM DMDC have 65- and 67-fold increases in intracellular copper, respectively, compared to the untreated control. Additionally, there was no statically significant difference in intrabacterial concentrations of zinc, manganese, or calcium for any condition comparison. Figure S2B shows a table of the means ± standard deviations (SD) for the experiments. Given these findings, these data suggest that treatment with DMDC and copper leads to increased intracellular copper concentrations and, thus, an increase in copper stress experienced by the bacterium.

### DMDC’s copper-dependent toxicity can be rescued by manganese supplementation.

We previously reported that DMDC exacerbates mismetallation as a mechanism of action via the addition of manganese rescuing DMDC’s copper-dependent toxicity *in vitro* (43). To directly test if copper-dependent toxicity associated with DMDC can be rescued by manganese supplementation after DMDC and copper addition, we performed a killing curve in which manganese was added at the 30-min time point (Fig. S2). While the addition of 500 μM Mn^2+^ at the 30-min time point rescued the 250 μM Cu^2+^ plus 16 μM DMDC condition, it did not rescue the 250 μM Cu^2+^ plus 32 μM DMDC condition (Fig. S1). Thus, the ability to rescue copper-dependent toxicity mediated by mismetallation exists as expected but with limitations. Nevertheless, these data provide further evidence for DMDC exacerbating copper-dependent mismetallation toxicity within the bacterium.

### DMDC- and copper-treated TIGR4 S. pneumoniae bacteria are killed at a higher rate by J774A.1 murine macrophages than are untreated bacteria.

**FIG 3:**
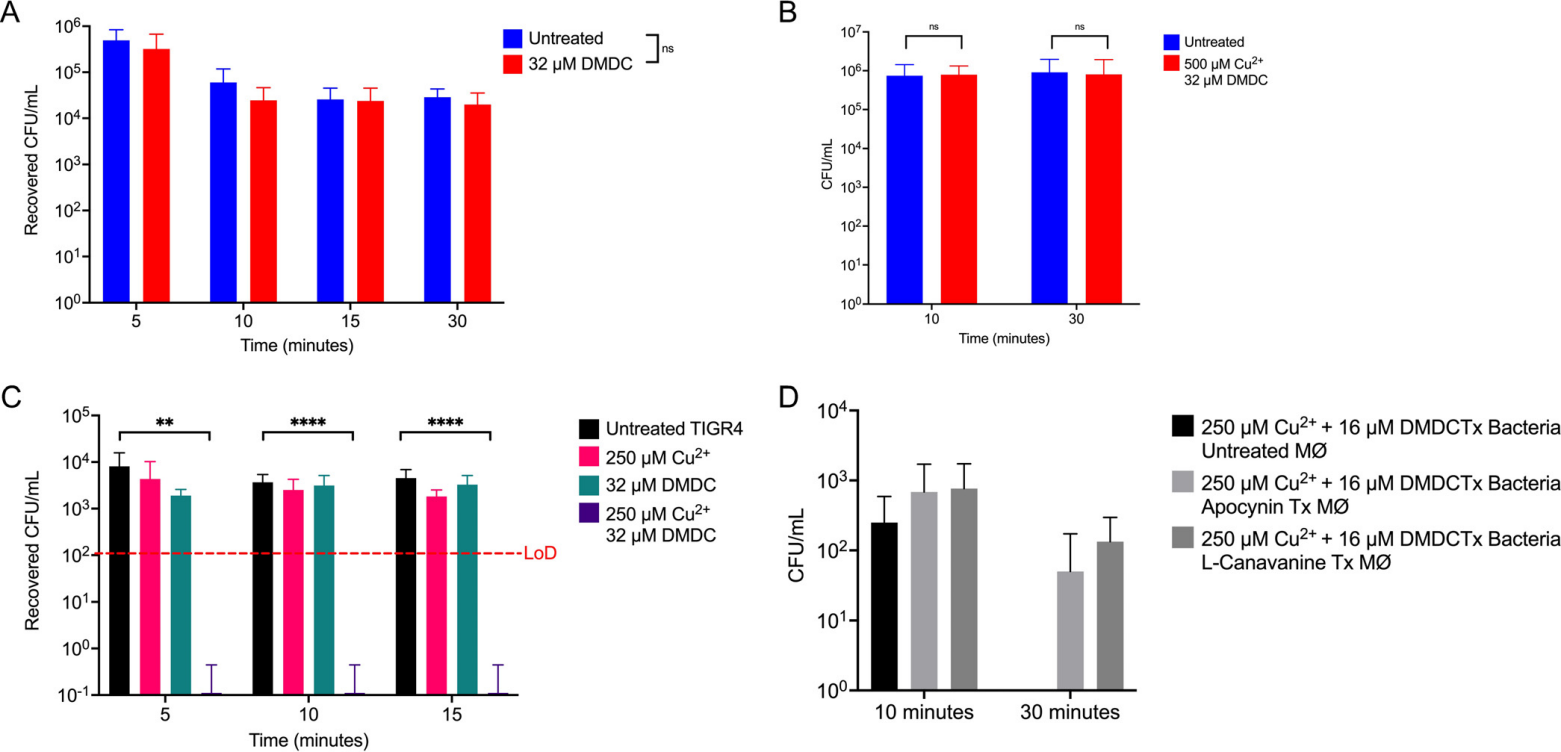
J774A.1 macrophages display enhanced *post hoc* killing of DMDC- and Cu^2+^-treated TIGR4 bacteria. (A) Macrophage killing assay of WT TIGR4 bacteria cocultured with activated J774A.1 macrophages. The initial inoculum of bacteria given to macrophages was 6.4 × 10^8^ CFU/mL for an MOI of 10. No statistically significant differences in killing rates or recovered CFU per milliliter were observed between untreated and 32 μM DMDC-pretreated macrophages. (B) Macrophage killing assay of WT TIGR4 bacteria as described above for panel A, with an initial inoculum of 9.2 × 10^6^ CFU/mL for an MOI of 10. No statistically significant difference in killing rates or recovered CFU/mL were observed between untreated and combination-pretreated macrophages. (C) Macrophage killing assay of WT TIGR4 bacteria cocultured with activated J774A.1 macrophages given bacteria that were treated with the indicated combinations of Cu^2+^ and DMDC. The initial inoculum of bacteria given to macrophages was 7.3 × 10^6^ CFU/mL (following a 15-min incubation under the indicted conditions) for an MOI of 10. There is a statically significant decrease in recovered CFU per milliliter between the untreated bacteria and Cu^2+^- and DMDC-treated bacteria at the 5-min time point. At this time point, all combination-treated bacteria were cleared by the macrophages, indicating a rapid *post hoc* bactericidal killing capacity. (D) Macrophage (MØ) killing assay of WT TIGR4 bacteria as described above for panel C, with an initial inoculum of 9.6 × 10^6^ CFU/mL for an MOI of 10. No statistically significant difference in *post hoc* recovery rates was observed between the conditions; however, there was a trend for improved recovery under the macrophage inhibitor treatment (Tx) conditions. All bars represent means ± SD (*n* = 9 to 12 across 3 independent replicates). Statistical differences were measured by Student’s *t* test (ns, not significant; *, *P* < 0.05; **, *P* < 0.01; ***, *P* < 0.001; ****, *P* < 0.0001).

Since the copper-dependent toxicity of DMDC is enhanced by incubation in a host-niche-mimicking medium (Fig. S1C and D), and macrophage-mediated clearance is a key mechanism of innate immune clearance of pathogenic S. pneumoniae, we wanted to directly test if *in vitro* incubation with murine macrophages leads to enhanced macrophage bactericidal activity. First, we determined if DMDC is cytotoxic to macrophages. We exposed J774A.1 macrophages to our highest used DMDC concentration and found that it was not toxic via a trypan blue cytotoxicity assay (Fig. S3). From there, we wanted to test if DMDC works by priming macrophages for improved killing. We treated macrophages with 32 μM DMDC while activating the macrophages (treatment with lipopolysaccharide [LPS] and IFN-γ) 6 h before coculturing with S. pneumoniae. We show no statistical difference between the recovered CFU per milliliter of bacteria from coculturing with the treated macrophages and the untreated control (Fig. 3A). We further tested if adding increased copper was necessary along with DMDC exposure to generate an improved killing rate. Incubation of macrophages with the combination of 500 μM Cu^2+^ plus 32 μM prior to coculture with S. pneumoniae did not show a significant difference in the killing rate compared to untreated control macrophages (Fig. 3B).

Next, we tested if DMDC can increase the efficiency of *post hoc* killing of antibiotic-treated bacteria by macrophages. We hypothesized that macrophages would kill bacteria given a moderate dosage of copper plus DMDC much faster than untreated, copper-alone, or DMDC-alone controls. In M17, all bacteria were still viable at 15 min at our highest dosages of DMDC plus copper (data not shown). Thus, we incubated the bacteria treated with 250 μM Cu^2+^ plus 32 μM DMDC for 15 min prior to coculturing with macrophages for 5-, 10-, and 15-min incubations to test macrophage killing ability. The bacteria recovered at each time point for the DMDC plus copper treatment were below the limit of detection (LoD) (Fig. 3C). As such, the recovered CFU counts show a statistically significant difference between the untreated and copper- and DMDC-treated groups. Overall, these data indicate that DMDC aids in the macrophage killing rate *post hoc* of copper- and DMDC-treated bacteria.

**FIG 4:**
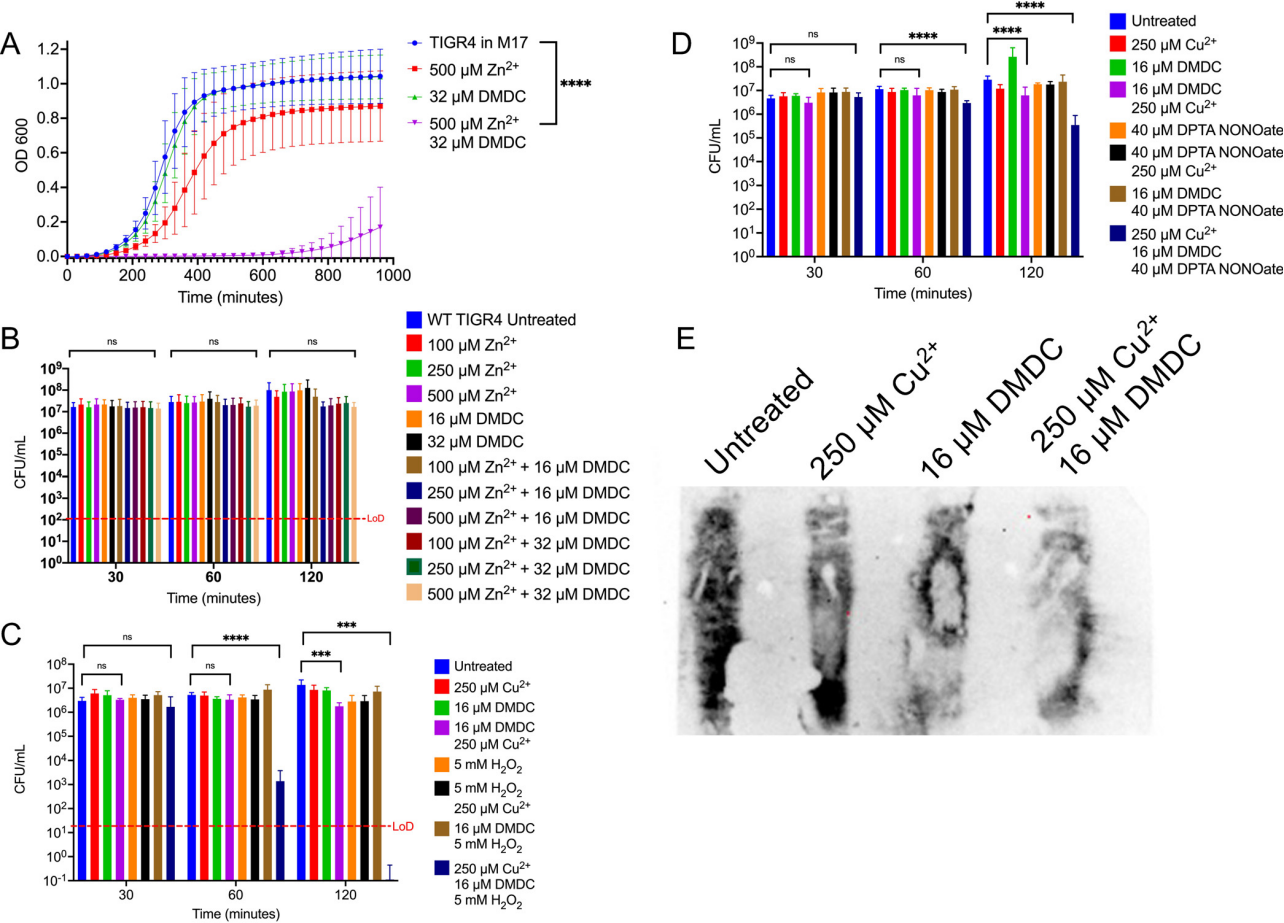
Mechanisms utilized by the macrophage phagolysosome synergize with DMDC’s copper-dependent toxicity. (A) Growth curve of WT TIGR4 S. pneumoniae in M17 medium supplemented with the indicated concentrations of zinc and/or DMDC, demonstrating a significant growth defect for the combination of 500 μM Zn^2+^ plus 32 μM DMDC. (B) Killing curve assay of WT TIGR4 starting with an inoculum of 1 × 10^7^ CFU/mL in M17 medium supplemented with a titration of combinations of zinc with or without DMDC, showing that the 500 μM Zn^2+^ plus 32 μM DMDC condition is bacteriostatic, with no statistically significant difference in CFU per milliliter for the two compared conditions. (C) Killing curve assay of WT TIGR4 starting with an inoculum of 4.0 × 10^6^ CFU/mL. S. pneumoniae was incubated in M17 medium supplemented with combinations of copper, DMDC, and hydrogen peroxide. Utilizing a smaller amount of copper (250 μM, compared to 500 μM used in previous figures), a smaller amount of DMDC (16 μM, compared to 32 μM utilized in previous figures), and a moderate amount of hydrogen peroxide (5 mM), to which S. pneumoniae TIGR4 is resistant, the combination of 5 mM H_2_O_2_ plus 250 μM Cu^2+^ and 16 μM DMDC displayed robust killing at the 60-min time point that extended to the 120-min time point. (D) Killing curve assay of WT TIGR4 starting with an inoculum of 6.0 × 10^6^ CFU/mL. S. pneumoniae was incubated in M17 medium supplemented with combinations of copper, DMDC, and DPTA NONOate, a nitric oxide-donating compound. The combination of 40 μM DPTA NONOate plus 250 μM Cu^2+^ and 16 μM DMDC displayed statistically significant killing at the 60- and 120-min time points. (E) Capsule blot of WT TIGR4 treated with the indicated concentrations of copper and DMDC, showing a decrease in capsule under the combination treatment condition. All bars for killing curves represent means ± SD (*n* = 9 across 3 independent replicates). Statistical differences were measured by Student’s *t* test (ns, not significant; *, *P* < 0.05; **, *P* < 0.01; ***, *P* < 0.001; ****, *P* < 0.0001). The capsule blot is representative of results from 3 independent replicates.

To test if the improvement in macrophage *post hoc* bacterial clearance is mediated by macrophage phagolysosomal nitric oxide and reactive oxygen species, we utilized inhibitors of these killing mechanisms in Fig. 4D. Macrophages were incubated with 100 μM apocynin to inhibit oxidative killing (52, 53). Alternatively, macrophages were incubated with 100 μM l-canavanine to inhibit nitric oxide (54, 55). Treated and untreated macrophages were given bacteria treated with 250 μM Cu^2+^ plus 16 μM DMDC (this was lower than the levels used in Fig. 3C). There was no statistically significant improvement in recovered bacteria with the macrophage treatment; however, there was a trend toward an improvement. Taken together, these mechanisms may partially contribute to the improved *post hoc* bacterial clearance.

### *In vitro* incubation of DMDC under conditions replicating the macrophage phagolysosome displays enhanced susceptibility to copper-dependent toxicity.

To mediate the killing of pathogens within the phagolysosome, macrophages utilize high concentrations of zinc, hydrogen peroxide, nitric oxide, and copper as well as having a low pH and proteases (15, 56, 57). To further understand why the pretreatment of bacteria led to rapid J774A.1 macrophage clearance of bacteria, we performed growth curve and killing curve assays *in vitro* to mimic the environment of the macrophage phagolysosome. We performed a growth curve with TIGR4 S. pneumoniae in M17 medium supplemented with the indicated combinations of zinc and DMDC and found that there is a growth defect under the 500 μM Zn^2+^ plus 32 μM DMDC condition (Fig. 4A). We also performed a killing curve in M17 medium with a titration of combinations of zinc with or without DMDC, showing that the 500 μM Zn^2+^ plus 32 μM DMDC condition is bacteriostatic: there is no significant difference in CFU per milliliter between the untreated control and the combination treatment (Fig. 4B).

To test the contribution of hydrogen peroxide to the macrophage killing seen in Fig. 4B, we performed a killing curve in which M17 medium was supplemented with combinations of copper, DMDC, and hydrogen peroxide (Fig. 4C). Utilizing a smaller amount of copper (250 μM, compared to 500 μM used in previous figures), a smaller amount of DMDC (16 μM, compared to 32 μM utilized in previous figures), and 5 mM hydrogen peroxide, with which S. pneumoniae TIGR4 displays control levels of growth, the combination of 5 mM H_2_O_2_ plus 250 μM Cu^2+^ and 16 μM DMDC displayed robust killing at the 60-min time point that extended to the 120-min time point (Fig. 4C).

To test the contribution of nitric oxide to the macrophage killing seen in Fig. 3B, we performed a killing curve by supplementing M17 medium with combinations of copper, DMDC, and a nitric oxide-donating compound, DPTA (diethylene triamine pentaacetic acid) NONOate (58–60). Macrophages produce around 40 μM nitric oxide when activated by IFN-γ and LPS (61). For this reason, we performed the killing curves with combinations of 40 μM DPTA NONOate in Fig. 4D. The combination of 40 μM DPTA NONOate plus 250 μM Cu^2+^ and 16 μM DMDC displayed statistically significant killing at the 60-min and 120-min time points compared to both the control and DMDC- and copper-treated bacteria. Overall, these data show that high zinc concentrations, hydrogen peroxide exposure, and nitric oxide exposure combine with the copper-dependent toxicity of DMDC to explain the rapid killing of bacteria when cocultured with macrophages *in vitro*.

### Combination treatment with DMDC and Cu^2+^ leads to a decrease in the amount of extracellular capsule of S. pneumoniae.

The extracellular polysaccharide capsule of S. pneumoniae is a well-characterized mechanism against innate immune cell phagocytosis and against macrophage-mediated killing. This role of the type 4 TIGR4 capsule has been confirmed by Rukke et al. (62). Hydrolysis of the capsule of type 3 S. pneumoniae renders the bacteria more susceptible to phagocytosis by macrophages and complement-mediated killing by neutrophils (63). Following established protocols for blotting against the type 4 capsule of the TIGR4 strain of S. pneumoniae (19), we show in Fig. 4E that the combination treatment of DMDC plus Cu^2+^ leads to a decrease in observed capsule in the pellet fraction of bacteria. There was no capsule observed in the supernatant (data not shown). These data provide evidence that the enhanced macrophage killing due to treatment with DMDC plus Cu^2+^ seen in Fig. 3B is due to a decrease in bacterial capsule that potentially allows increased macrophage opsonization.

### *In vivo* treatment with DMDC increases lung macrophage and dendritic cell populations upon S. pneumoniae infection.

**FIG 5:**
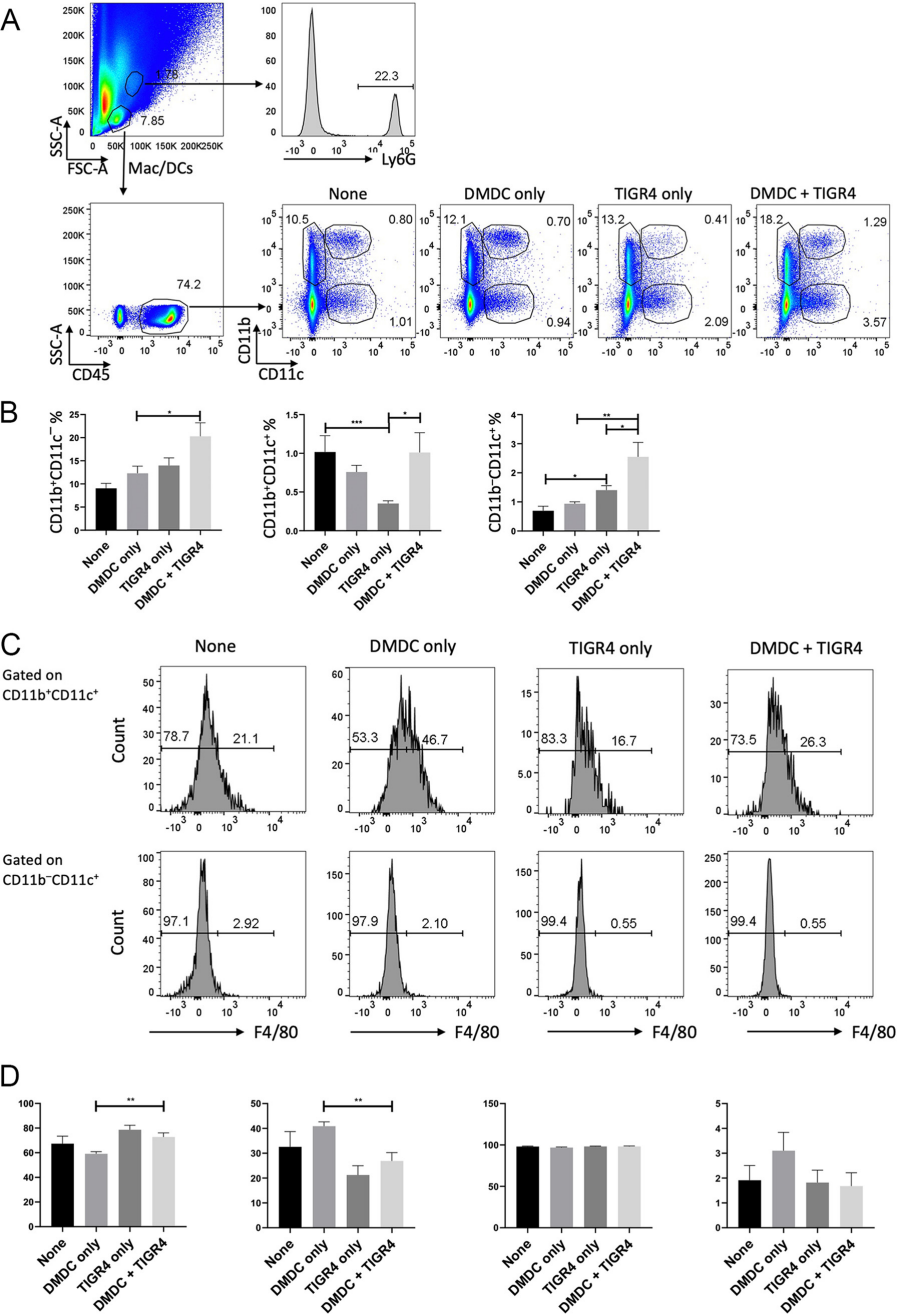
Effect of DMDC treatment on macrophage and DC populations in the lungs of BALB/c mice infected with TIGR4. Groups of 8-week-old mice were either untreated (none), given the compound DMDC (DMDC only), infected with TIGR4 intranasally (TIGR4 only), or treated with DMDC 8 h after TIGR4 infection (DMDC + TIGR4). (A) Representative percentages of Ly6G^+^ neutrophils in untreated mice and CD11b^+^ CD11c^−^, CD11b^+^ CD11c^+^, and CD11b^−^ CD11c^+^ cells from CD45^+^ leukocytes of each group. (B) Quantitative percentages of leukocyte populations in panel A shown as means and standard errors of the means (SEM) (*n* = 3 for the untreated group, and *n* = 10 for all other groups [from three assays combined]). (C) Representative histograms of percentages of F4/80^−^ and F4/80^+^ cells from CD11b^+^ CD11c^+^ and CD11b^−^ CD11c^+^ populations in panel A. (D) Quantitative percentages of F4/80^−^ and F4/80^+^ cells in panel C. Statistical differences were measured by a two-tailed, unpaired *t* test with Welch’s correction (ns, not significant; *, *P* < 0.05; **, *P* < 0.01; ***, *P* < 0.001; ****, *P* < 0.0001).

In our laboratory’s previous study identifying DMDC’s copper-dependent antibiotic effect, treatment with DMDC alone significantly decreased the bacterial burden in a murine pneumonia model of infection (43). Here, we focused on the *in vivo* effect of DMDC on the macrophage and dendritic cell (DC) populations of mice treated with DMDC. Cohorts of 8-week-old female BALB/cJ mice were treated under four conditions: (i) no treatment, (ii) DMDC only, (iii) TIGR4 only, and (iv) DMDC and TIGR4. Lung single-cell suspensions were stained and gated on the indicated forward-scatter (FSC)/side-scatter (SSC) areas to avoid the cell population enriched with infiltrating Ly6G-positive (Ly6G^+^) neutrophils (Fig. 5A). The cells were further gated on CD45 for leukocytes and then CD11b versus CD11c, which can group cells into 3 subsets: (i) CD11b^+^ CD11c^−^, (ii) CD11b^+^ CD11c^+^, and (iii) CD11b^−^ CD11c^+^ populations (Fig. 5A). Group 1 is enriched with monocytes and a few neutrophils, group 2 is enriched with interstitial macrophages, and group 3 contains both alveolar macrophages and dendritic cells (64). We saw significant increases in group 2 CD11b^+^ CD11c^+^ and group 3 CD11b^−^ CD11c^+^ populations in DMDC- and TIGR4-treated lungs compared to the TIGR4-only-treated group (Fig. 5B). As seen in group 2, DMDC treatment seemed to restore the interstitial macrophage population to wild-type (WT) levels as it was reduced under the TIGR4-alone condition. We further gated group 3 into F4/80^+^ and F4/80^−^ groups for alveolar macrophages and DCs, respectively, and found that unlike group 2 CD11b^+^ CD11c^+^ cells, group 3 CD11b^−^ CD11c^+^ cells were mostly F4/80 negative and thus were enriched with dendritic cells (Fig. 5C and D). Overall, these data show that DMDC treatment, through interaction with either the bacteria or the immune system, resulted in more immune cells in the lung environment, which corresponded with the reduction of the bacterial burden *in vivo*.

## DISCUSSION

Clinically, it is difficult to untangle if an antibiotic works to increase macrophage clearance of pathogens by working with macrophages directly or on bacteria to allow macrophage *post hoc* clearance. We tested this hypothesis by first finding that DMDC works on the pneumococcus *in vitro* independent of macrophages (see Fig. S1 in the supplemental material) before using coculture experiments to answer this question for DMDC directly (Fig. 3). Here, we showed that DMDC treatment with copper sensitizes the pneumococcus to macrophage killing by increasing the internal copper concentration (Fig. 2 and Fig. S2), increasing susceptibility to the phagolysosomal weapons H_2_O_2_ and nitric oxide (Fig. 4C and D), and decreasing the size of the antiphagocytic pneumococcal capsule (Fig. 4E). We further demonstrated that the *in vivo* administration of DMDC to mice upon pneumococcal infection increases the lung myeloid cell populations within the lung, corresponding to the reduction of the bacterial burden. While changing the environment of the bacteria away from the DMDC plus copper condition allows the bacteria to eventually begin recovery (Fig. 1), the inadequate replenishment of environmental nutrients under significant stress quickly led to a reduction in the capsule. Due to the antiphagocytic nature of the bacterial capsule, we believe that it is reasonable to postulate that the increases in macrophage-mediated pneumococcal killing and the numbers of macrophages and dendritic cells within the pneumococcus-infected lung during DMDC treatment after pneumococcal infection are due to the culmination of these DMDC-mediated effects.

While how DMDC facilitates the increased influx of intrabacterial copper remains a mystery, it is abundantly clear that treatment with DMDC plus copper leads to significant increases in internal copper in the pneumococcus. To this end, given the level of copper that we used in our assays, the amount of internal copper with the combination treatment compared to copper alone with the pneumococcus and bacteria in other studies is unparalleled. This result represents increased opportunities where DMDC and other successfully tested and verified ionophores can be used in addition to copper to illicit a -static or -cidal effect on invading pathogens.

Increases in intracellular copper concentrations were not linear, suggesting that a threshold for either DMDC-copper import or copper export kinetics prevents higher accumulation of copper. Further adding to the complexity of a potential mechanism for import is that there is no known import system for copper in the pneumococcus. Siderophores and zincophores can bind to copper, facilitating active import incidentally (65, 66). Therefore, although their structures vary greatly, one possibility is that DMDC mimics an ionophore that the pneumococcus readily takes up, thus leading to copper’s increased import. If copper-dependent toxicity therapeutics are to be developed, future studies will need to determine metal import and export kinetics with respect to known organismal influx and efflux systems and those yet to be discovered. These rates could ultimately determine the effectiveness of DMDC and other similar compounds using copper-dependent toxicity against a given pathogen.

While subject to future studies, we hypothesize based on this study that DMDC and copper treatment supports a new mechanism of copper toxicity for some bacteria. In the assays for our growth and killing curves, in the lung environment from surrounding copper-bound proteins, and in the phagolysosome, exists an oxidizing environment for copper. Thus, the pneumococcus in these environments must process copper in its oxidized Cu^2+^ form but, as defined by the literature and CopA homology, export copper in the reduced Cu^1+^ form (67–70). Thus, the necessity to reduce copper would require a constant supply of electrons to facilitate export through the CopA exporter. A common source of electrons is from reducing sugars, proven *in vitro*, as shown by Benedict’s test (71, 72). Here, copper is reduced when combined with sodium citrate, sodium carbonate, and a sugar that contains a ketone or aldehyde. S. pneumoniae upregulates sugar import systems under conditions of copper stress; one specific importer of interest to focus future studies on is SP0478, encoded by the *lacE* gene (73). Furthermore, bacteria contain invertases that can convert nonreducing sugars to a reducing sugar and water. While it might not be needed and thus not observed in complex media plus copper due to abundant resources, DMDC plus copper induced higher levels of internal copper than in previous reports, and minimal environmental (e.g., host) resources could conceivably elicit a need for electrons so great as to force the bacteria to take up any available sugars, including those contained within their extracellular polysaccharide capsule.

Taken together, while bacteria struggle with mismetallation due to copper stress, they must also battle to eliminate the threat by exporting copper. In oxidizing environments, bacteria reducing copper for export comes at the cost of their reducing environment, which, if not replenished, will lead to dire consequences. While bacteria can import environmental carbohydrates, the nearest source for the pneumococcus is the capsule. The capsule is protection against future threats such as the macrophage; however, the immediate threat of increasing the ability to export copper is the more pressing issue for survival. Thus, the pneumococcus cannibalizes its capsule for nutrients, making it far more susceptible to macrophage recognition (and, therefore, recruiting other immune cells), opsonization, and, ultimately, killing mechanisms.

## MATERIALS AND METHODS

### Bacterial culture.

M17 medium (BD Difco, USA) was prepared according to the manufacturer’s instructions. Briefly, 37.25 g of powder was suspended in 950 mL of MilliQ-grade water (≥18.0 MΩ cm^−1^) and autoclaved at 121°C for 15 min before cooling to 50°C and adding 50 mL of a sterile 10% lactose solution. Gibco RPMI 1640 medium containing l-glutamine and 4 g/L NaHCO_3_ was purchased from the University of Arizona BIO5 Institute Media Facility. Prior to growth in RPMI 1640, cold RPMI 1640 was supplemented with 0.1 mg/mL catalase, 30 mM glucose, 1× trace metals, and 1× “supplements” as described previously by Schulz et al. and further delineated by Sanchez-Rosario and Johnson (48, 74). Tryptic soy agar (TSA) (Hardy Diagnostics, USA) was dissolved in MilliQ water and autoclaved. After cooling autoclaved TSA, a 5% final volume of defibrillated sheep’s blood (HemoStat Laboratories) and 20 μg/mL neomycin were added to the solution. These plates (blood agar plates [BAPs]), were used for routine culture on solid media and for “killing curve” serial dilution CFU counting. Bacteria from freshly streaked plates were placed into M17 and grown at 37°C in 5% CO_2_ to an optical density (OD) (or OD at 600 nm [OD_600_]) of 0.125 for growth curve assays and to an OD of ∼0.300 for killing curve assays. To prepare working stocks of viable S. pneumoniae, growing cultures were resuspended in fresh medium with 20% (vol/vol) glycerol and stored at −80°C. Aliquot viability and CFU counts were determined as discussed below before use in experiments. Glycerol stock aliquots were diluted 1:5 into M17 or RPMI 1640 with the indicated copper and compound concentrations for assays.

### Growth curves.

Copper stock solutions at 100 mM were prepared from CuSO_4_ pentahydrate (VWR Life Sciences, USA) in MilliQ water. Stock solutions of 100 mM Zn^2+^ were prepared from ZnSO_4_ heptahydrate (VWR Life Sciences, USA). Stock solutions of 100 mM DMDC were prepared from sodium dimethyldithiocarbamate dihydrate (Tokyo Chemical Industry, Japan) in MilliQ water. Sterile, individually wrapped, clear 96-well polystyrene plates (Greiner Bio-One, USA) were arranged to test a range of concentration combinations of Cu^2+^, Zn^2+^, and DMDC. Frozen aliquots of S. pneumoniae were thawed and diluted 5-fold into fresh M17 before adding 20 μL per well into a total well volume of 200 μL (1:50 total dilution). Assay plates were loaded into a Cytation5 instrument (BioTek, VT, USA) preequilibrated to 37°C with 4% CO_2_. Gas control settings were modified for an elevation of 720 m according to the manufacturer’s directions. The Cytation5 assay protocol maintained temperature and CO_2_ while measuring the OD at 600 nm every 30 min for 16 to 20 h.

### Killing curves.

Aliquots of S. pneumoniae were thawed and diluted 10-fold into assay mixtures prepared in M17 or RPMI 1640. Assay conditions included various concentrations of CuSO_4_, DMDC, hydrogen peroxide (Sigma-Aldrich), and DPTA NONOate (Cayman Chemical Company, USA). After exposure to the indicated conditions, bacteria were incubated at 37°C in 5% CO_2_ for the indicated times, and samples were serially diluted, plated onto BAPs, incubated overnight at 37°C in 5% CO_2_, and counted to determine viable CFU unless variations are otherwise specified in the specific figure legends. Colonies on each plate were counted and multiplied by the appropriate dilution factor based on which dilution it was to determine CFU per milliliter. For plates in which no colonies were visualized at all, they were deemed to be below the limit of detection (LoD) and are noted with a data point below the LoD line.

### Graphite furnace atomic absorption spectroscopy.

Graphite furnace atomic absorption spectroscopy (GFAAS) experiments were performed in triplicate. TIGR4 S. pneumoniae cells were initially cultured on M17 plus 5 mM lactose and frozen at −80°C in 20% glycerol. These glycerol stocks were used as the seed stocks to inoculate 40 mL of M17 plus 5 mM lactose. The bacterial culture was incubated at 37°C under 5% CO_2_ until an OD of ∼0.300 was reached. The culture was split into the indicated treatment and control groups. Incubation for treatments was performed at 37°C with 5% CO_2_ for 30 min. Samples were quenched in a −3°C water bath to slow down metabolism, followed by 2 washes with cold Tris-buffered saline (TBS) (50 mM Tris, 150 mM NaCl, and 50 mM EDTA at pH 7.6) and centrifugation at 7,800 × *g* for 7 min at 4°C. Cold decanted samples were stored at −20°C overnight before resuspension in 5% HNO_3_. Bacterial plate counts were performed on TSA plus 5% sheep’s blood through serial dilutions, as described above. Samples were analyzed for copper content using a Thermo iCE 3400 atomic absorption spectrometer with a 324.8-nm wavelength. Standards were made using the TraceCERT copper standard for AAS from Sigma-Aldrich, and the copper content of the washed samples was calculated based on the average absorbance from at least six independent measurements with four resamplings each. Baseline measurements of media alone were conducted to ensure instrument accuracy, finding copper levels to be consistent with those of the untreated control. Statistical significance was determined by an unequal-variance *t* test, with *P* values indicated in the figures (*, *P* < 0.05; **, *P* < 0.01; ***, *P* < 0.001; ****, *P* < 0.0001).

### Capsule blotting.

The protocol used for capsule blotting was adapted from methods described previously by Kietzman et al., with modifications (19). Briefly, bacteria from freshly streaked BAPs were grown in M17 medium to an OD of ∼0.400 prior to separating the bacteria into 1-mL cultures and exposing them to the indicated conditions for 30 min. Equal CFU per milliliter were obtained under each condition. Following exposure, bacteria were pelleted by centrifugation at 3,500 × *g* for 10 min and resuspension of the pellet in 1 mL of SMH buffer (0.5 M sucrose, 0.02 M MgCl_2_, and 0.02 M HEPES). Next, bacterial pellets were centrifuged at 14,000 × *g* and treated with 100 μL of 10-mg/mL lysozyme (Gold Biotechnology, USA) and 20 μL of proteinase K (Gold Biotechnology, USA) at room temperature for 10 min. Pellets were then exposed to 13 μL of 10× SDS buffer and boiled for 10 min at 95°C, and 20 μL of each sample was loaded onto a 0.8% agarose gel. Samples were transferred onto a mixed nitrocellulose ester membrane via 20× SSC (1× SSC is 0.15 M NaCl plus 0.015 M sodium citrate) capillary transfer overnight. Membranes were cross-linked at 150,000 mJ using a Stratagene UV cross-linker, blocked for 1 h in phosphate-buffered saline (PBS)–Tween (PBST) with milk, probed with a 1:1,000 dilution of anticapsular antiserum (serotype 4, catalog number 16747; SSI Diagnostica), washed with 1× PBST for 5 min 3 times, probed with a 1:30,000 dilution of secondary antibody (horseradish peroxidase conjugated), washed again in 1× PBST for 5 min 3 times, and imaged on an imager following the addition of ECL reagent (Cytiva, USA) as specified by the manufacturer.

### Macrophage killing assays.

J774A.1 macrophages (ATCC, USA) were maintained in a 37°C incubator with 5% CO_2_ in Dulbecco’s modified Eagle’s medium (DMEM; Sigma-Aldrich, USA) containing fetal bovine serum (FBS) (10% [vol/vol]; Sigma-Aldrich, USA), glutamine (2 mM; Sigma-Aldrich, USA), penicillin (50 U/mL; Sigma-Aldrich, USA), streptomycin (50 mg/mL; Sigma-Aldrich, USA), and NaHCO_3_ (0.015%). Cells were grown to 90% confluence in 12-well tissue culture plates (Greiner CellStar; Greiner Bio-One, USA) in 1 mL/well of growth medium. On the morning of the experiment, macrophages were washed twice with 1 mL PBS and resuspended in 1 mL of serum-free DMEM growth medium without antibiotics, glutamine, NaHCO_3_, or FBS but supplemented with 5 ng/mL IFN-γ (Bio Basic, USA) and 400 ng/mL LPS (EMD Millipore, MilliporeSigma, USA) for the “priming of macrophages” experiment. For experiments involving treatment with apocynin (Santa Cruz Biotechnology, USA) or l-canavanine (Sigma-Aldrich, USA), serum-free DMEM was supplemented with 100 μM each inhibitor, as indicated. For the *post hoc* killing efficiency experiment, macrophages were resuspended in 1 mL of serum-free DMEM containing 32 μM DMDC, 5 ng/mL IFN-γ, and 400 ng/mL LPS.

Macrophages were incubated at 37°C with 5% CO_2_ for 12 h. Glycerol stocks of TIGR4 S. pneumoniae bacteria kept at an OD of 0.3 were removed from −80°C storage, diluted into four 15-mL conical tubes containing 5 mL total M17 plus lactose containing no additives (“untreated”), 32 μM DMDC, 250 μM CuSO_4_, and 250 μM CuSO_4_ plus 32 μM DMDC, respectively, for the experiment with pretreatment of bacteria. Prior to incubation of bacteria at 37°C with 5% CO_2_, an inoculum plate was made by serially diluting 100 μL from the no-additive conical tube. After 15 min of incubation, bacteria were centrifuged at 4,500 × *g* for 10 min and resuspended in DMEM without antibiotics, glutamine, NaHCO_3_, or FBS. Macrophages were removed from incubation, medium was removed, and cells were washed with 1 mL PBS twice and then infected with 100 μL of S. pneumoniae solutions for both experiment types, corresponding to a multiplicity of infection (MOI) of 10 bacteria per macrophage. The 12-well tissue culture plates were centrifuged at 200 × *g* for 2 min to facilitate coculturing.

Wells were then washed twice with PBS at the indicated time points; each wash was followed by a 5-min incubation in a 37°C, 5% CO_2_ incubator in DMEM containing gentamicin (50 μg/mL). Macrophages were lysed in 0.02% SDS in double-distilled water (ddH_2_O) and serially diluted to determine the counts of viable intracellular bacteria. Data were normalized to the level of killing observed for the untreated TIGR4 bacteria for each assay.

### Animal experiments.

All mouse studies were conducted with prior approval and under the guidelines of the Institutional Animal Care and Use Committee at the University of Arizona (IACUC protocol number 18-410, R35 GM128653). All mice were maintained in a biosafety level 2 (BSL2) facility and monitored daily for signs of moribundity. Eight-week-old female BALB/cJ mice (Jackson Laboratory, USA) were anesthetized with 3% isoflurane and intranasally given either (i) 25 μL of TBS (50 mM Tris, 150 mM NaCl [pH 7.4]), (ii) 0.8 mg/kg of body weight of DMDC in 25 μL of TBS, (iii) an inoculum of 1 × 10^7^ CFU of viable S. pneumoniae bacteria in 25 μL of TBS, or (iv) 1 × 10^7^ CFU of viable S. pneumoniae bacteria in 25 μL of TBS and subsequently 0.8 mg/kg of DMDC in 25 μL of TBS. Control TBS and bacterial infections were carried out 8 h prior to mice being given DMDC. For group 4, mice were intranasally infected before being treated with DMDC approximately 8 h later. Mice were sacrificed by CO_2_ asphyxiation and immediately dissected for lung and blood collection 48 h after infection and treatment. Lung tissue was collected into 1.5-mL tubes containing 500 μL Dulbecco’s phosphate-buffered saline (DPBS; Gibco, USA). Single-cell suspensions were prepared from lung tissue as described below.

### Preparation of single-cell suspensions from the lung.

Single-cell suspensions were prepared as previously described by Felix et al. (75, 76). Briefly, lungs were perfused with PBS and finely minced before being placed into digestion buffer containing 1 mg/mL collagenase D (MilliporeSigma, Darmstadt, Germany) and 0.15 mg/mL DNase I (Sigma-Aldrich, USA) in DMEM (HyClone; Sigma-Aldrich, USA) (77–80). Lungs were digested for 20 to 25 min at 37°C at 200 rpm and then passed through a 40-μm cell strainer to prepare single-cell suspensions.

### Antibodies and flow cytometry.

For surface staining, fluorophore-conjugated monoclonal antibodies (mAbs) specific for CD11b (clone M1/70), CD45 (clone 30-F11), F4/80 (clone BM8), and Ly6G (clone 1A8) were obtained from BioLegend (USA), and fluorophore-conjugated mAb specific for CD11c (clone HL3) was obtained from BD Biosciences (USA). Cells were run on an LSRII instrument (BD Biosciences, USA), and analyses were performed with FlowJo software (TreeStar, BD Biosciences, USA).

### Statistical analysis.

Statistical significance was analyzed using Student’s *t* test (two tailed, unpaired), two-way analysis of variance (ANOVA), or one-way ANOVA with Dunnett’s multiple-comparison test (Prism 9.20; GraphPad Software, USA). The *P* values are indicated in the figures (*, *P* < 0.05; **, *P* < 0.01; ***, *P* < 0.001; ****, *P* < 0.0001).
