# Supplementary material for: N,N-Dimethyldithiocarbamate Elicits Pneumococcal Hypersensitivity to Copper and Macrophage-Mediated Clearance
Source: Infect Immun. 2022 Mar 21;90(4):e00597-21. doi: 10.1128/iai.00597-21 (PMC9022595; doi:10.1128/iai.00597-21)

Supplemental Data for *N,N*-dimethyldithiocarbamate elicits pneumococcal hypersensitivity to copper and macrophage-mediated clearance

Sanjay V. Menghani[1,2], Madeline P. Cutcliffe[1], Yamil Sanchez-Rosario[1], Chansorena Pok[1], Alison Watson[1], Miranda J. Neubert[1], Klariza Ochoa[1], Hsin-Jung Joyce Wu[1,3], Michael D. L. Johnson[1,4,5,6]

[1]Department of Immunobiology
University of Arizona College of Medicine - Tucson
Tucson, AZ, 85724
[2]Medical Scientist Training MD-PhD Program (MSTP)
University of Arizona College of Medicine - Tucson
Tucson, AZ, 85724
[3]Arizona Arthritis Center
University of Arizona College of Medicine - Tucson,
Tucson, AZ, 85724
[4]Valley Fever Center for Excellence
University of Arizona College of Medicine - Tucson
Tucson, AZ, 85724
[5]BIO5 Institute
University of Arizona College of Medicine - Tucson
Tucson, AZ, 85724
[6]Asthma and Airway Disease Research Center
University of Arizona College of Medicine - Tucson
Tucson, AZ, 85724

Corresponding Author: Michael D. L. Johnson
University of Arizona
1656 E. Mabel St. / P.O. Box 245221 / MRB 213 (office)
Tucson, AZ 85724
Tel: 520-626-3779 / Fax: 520-626-2100
mdljohnson@arizona.edu

Supplemental Methods

Inductively Coupled Plasma Optical Emission Spectroscopy

Experiments were performed in triplicate. TIGR4 *S. pneumoniae* were initially cultured on M17 + 5 mM lactose and frozen at -80°C in 20% glycerol. These glycerol stocks were used as the seed stock to inoculate 150 mL of M17 + 5 mM lactose. The bacterial culture was incubated at 37°C under 5% CO_2 until an OD of ~0.400 was reached. The culture was split into the indicated treatment and control. Incubation of treatments was performed at 37°C and 5% CO_2 for 30 minutes. Samples were quenched in -3°C water bath to slow down metabolism, followed by 2 washes of cold TBS (tris 50 mM, NaCl 150 mM, EDTA 50 mM at pH 7.6), and centrifugation 3500x g for 10 minutes at 4°C. Cold decanted samples were resuspended in 2% HNO_3. Bacterial plate counts were performed in TSA + 5% Sheep's Blood through serial dilutions, as described above. Samples were analyzed for metal content using an iCAP PRO XDUO ICPOES with a wavelength 324.8 nm copper, 213.8 nm for zinc, 257.6 nm for manganese and 393.3 nm for calcium. Standards were made using iCap Series Multi-element test solution ICAP 6000 series Validator from Thermo Scientific (Thermo Scientififc, USA) and metal content of the washed samples was calculated using the Qtegra software. Baseline measurements of media alone were conducted to ensure instrument accuracy, finding copper levels to be consistent with that of the untreated control.

Supplemental Figure Legends

Supplemental Figure 1 – Copper-dependent cytotoxicity of DMDC is enhanced in host-niche mimicking media in comparison to nutrient-rich media. (A) Growth curve of WT TIGR4 *S. pneumoniae* in M17 media supplemented with indicated concentrations of copper and/or DMDC, demonstrating a significant growth defect for the combination of 500 µM Cu^{2+} + 32 µM DMDC. **(B)** Killing curve assay of WT TIGR4 starting with an inoculum of $1x10^{7}$ CFU/mL in M17 media supplemented with indicated concentrations of copper and/or DMDC, demonstrating a significant decrease in CFU/mL to a level below our level of detection at t = 120-minutes for the 500 µM Cu^{2+} + 32 µM DMDC combination, indicating bactericidal activity. **(C)** Growth curve of WT TIGR4 in RPMI media supplemented with copper and/or DMDC, demonstrating a significant growth defect for the combination of 50 µM Cu^{2+} + 16 µM DMDC. **(D)** Killing curve assay of WT TIGR4 starting with an inoculum of $5.8x10^{6}$ CFU/mL in RPMI media supplemented with copper and/or DMDC, demonstrating a bactericidal combination of 250 µM Cu^{2+} + 16 µM DMDC at the t = 180 and t = 240-minutes timepoints. All bars for growth curves represent mean ± standard deviation (SD) with n = 18 across 3 independent replicates. Statistical difference measured by one-way ANOVA and Dunnett's multiple comparisons test; ns = not significant, *p < 0.05, **p < 0.01, ***p < 0.001, and ****p < 0.0001. All bars for killing curves represent mean ± standard deviation (SD) with n = 9 across 3 independent replicates. Statistical difference measured by Student's *t* test; ns = not significant, *p < 0.05, **p < 0.01, ***p < 0.001, and ****p < 0.0001.

Supplemental Figure 2 – ICP-OES analysis of DMDC + copper treatment on intra-bacerial zinc, manganese, copper, and calcium levels. (A) ICP-OES analysis of bacterial pellets showing a marked statistically significant increase in copper content within the bacterium of 250 µM Cu^{2+} + 16 µM DMDC-treated bacteria and within the bacterium of 250 µM Cu^{2+} + 32 µM

DMDC-treated bacteria in comparison to the untreated control. No significant increase was observed for intra-bacterial zinc, manganese, or calcium level for any experimental condition compared to control. **(B)** Mean ± standard deviation for the concentrations of each metal for the indicated experimental conditions. Experiments were performed in triplicate with statistical significance of differences determined through unequal variances *t* test; ns = not significant, *p < 0.05, **p < 0.01, ***p < 0.001, and ****p < 0.0001.

Supplemental Figure 3 – Manganese supplementation of DMDC + Cu^{2+}-treated *S. pneumoniae* can rescue toxicity to a threshold amount. Killing curve of WT TIGR4 *S. pneumoniae* in M17 media starting with an inoculum of 8.0×10^6 CFU/mL in M17 media supplemented with indicated concentrations of copper and/or DMDC for 30 minutes, at which point all conditions were supplemented with 500 µM Mn^{2+}. Manganese supplementation ablated the killing effect of 250 µM Cu^{2+} + 16 µM DMDC, but was not able to rescue the toxicity of 250 µM Cu^{2+} + 32 µM DMDC. Rescue is shown by a lack of statistically significant difference in CFU counts at t = 60-minutes and t = 120-minutes between Untreated and 250 µM Cu^{2+} + 16 µM DMDC conditions. All bars represent mean ± standard deviation (SD) with n = 9 across 3 independent replicates. Statistical difference measured by Student's *t* test; ns = not significant, *p < 0.05, **p < 0.01, ***p < 0.001, and ****p < 0.0001.

Supplemental Figure 4 – DMDC treatment is not cytotoxic to J774A.1 macrophages. Using a Trypan blue cytotoxicity assay, incubation of J77A.1 macrophages with up to 32 µM DMDC (the maximum dosage in our assays), did not display a significant difference in cellular viability after a 2-hour incubation. All bars represent mean live cell percentage ± standard deviation (SD) with n = 3 across 3 independent replicates. Statistical difference measured by Student's *t* test; ns = not significant; *p < 0.05, **p < 0.01, ***p < 0.001, and ****p < 0.0001.

Supplemental Figure 1

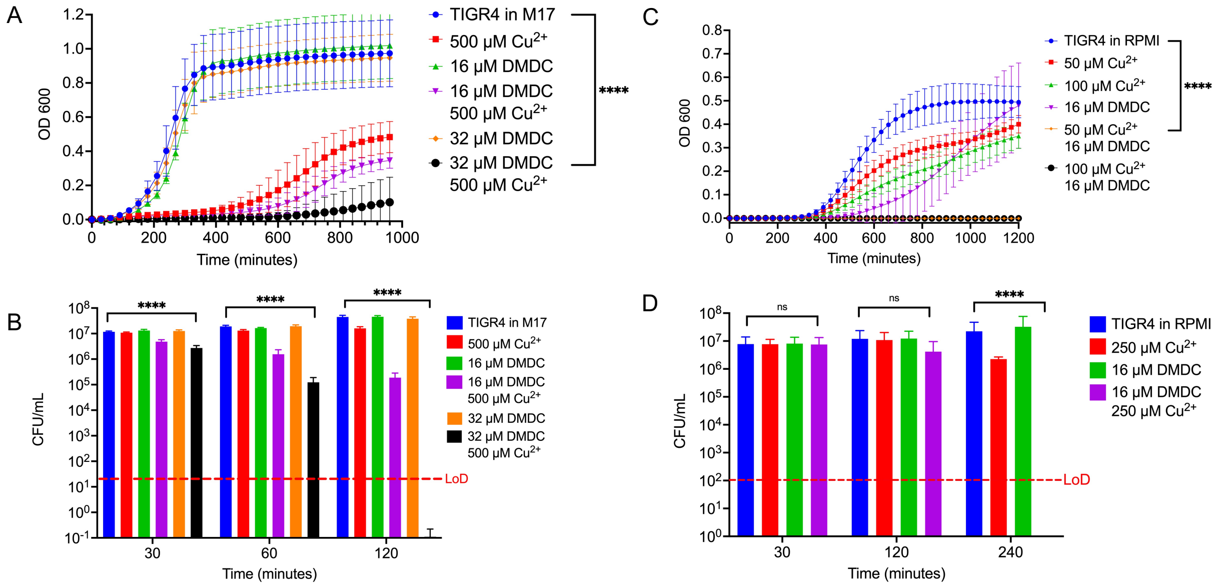

Supplemental Figure 2

A

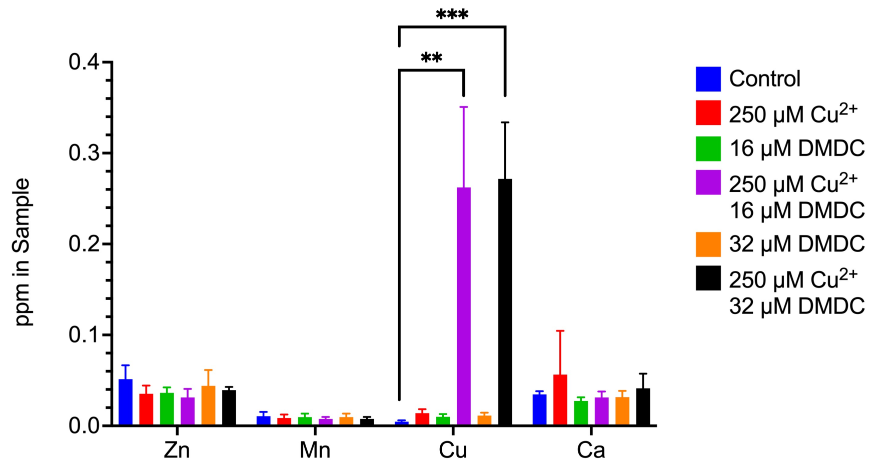

B

	Zn	Mn	Cu	Ca
**Control**	0.051 ±0.015	0.010 ±0.004	0.004 ±0.001	0.034 ±0.003
**250 µM Cu^{2+}**	0.035 ±0.009	0.008 ±0.003	0.014 ±0.004	0.056 ±0.048
**16 µM DMDC**	0.036 ±0.006	0.009 ±0.003	0.010 ±0.003	0.027 ±0.004
**250 µM Cu^{2+} 16 µM DMDC**	0.031 ±0.009	0.007 ±0.002	0.262 ±0.088	0.031 ±0.006
**32µM DMDC**	0.044 ±0.017	0.009 ±0.003	0.011 ±0.003	0.031 ±0.006
**250 µM Cu^{2+} 32 µM DMDC**	0.039 ±0.003	0.007 ±0.002	0.271 ±0.062	0.041 ±0.016

Supplemental Figure 3

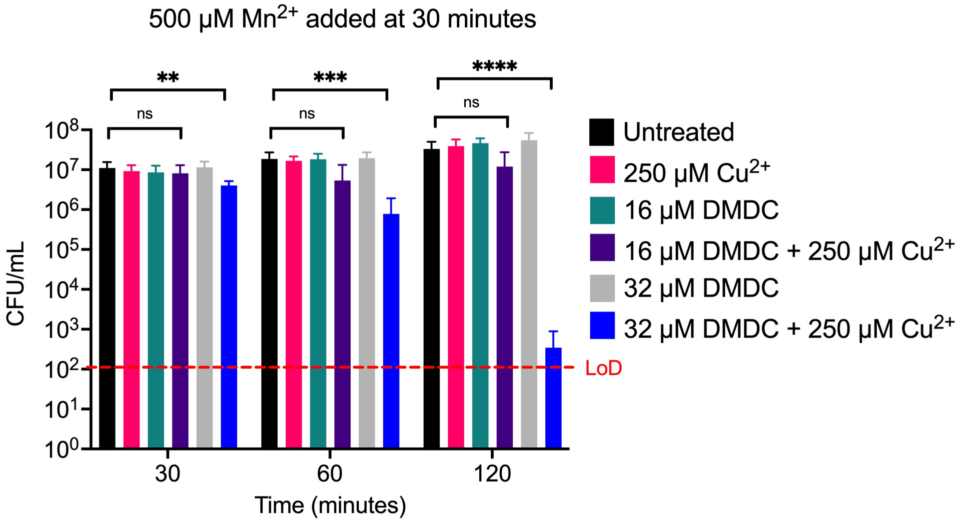

Supplemental Figure 4

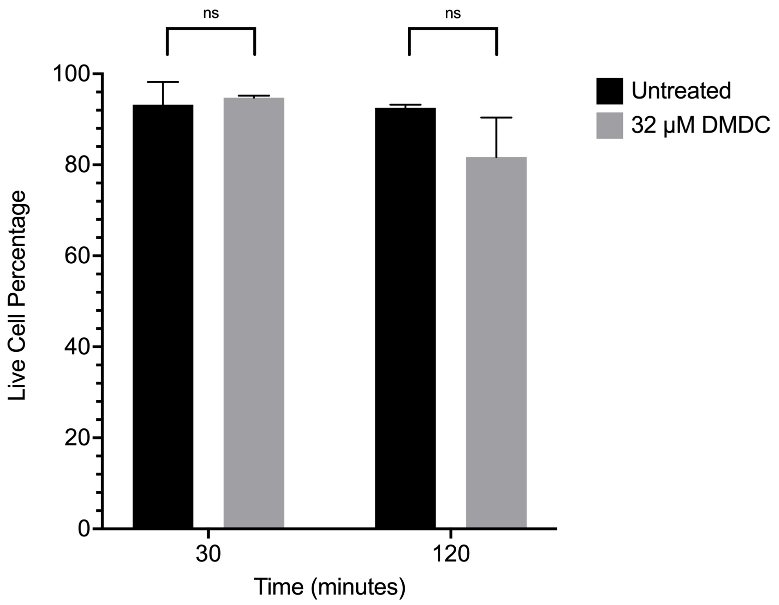

Supplement: Supplemental file 1 — Supplemental material. Download iai.00597-21-s0001.pdf, PDF file, 2.3 MB [file iai.00597-21-s0001.pdf]
